# *De Novo* Whole-Genome Sequencing and Annotation of Pathogenic Bovine Pasteurella multocida Type A:3 Strains

**DOI:** 10.1128/MRA.01078-20

**Published:** 2020-12-03

**Authors:** Morag Livingstone, Kevin Aitchison, Mark Dagleish, David Longbottom

**Affiliations:** a Moredun Research Institute, Pentlands Science Park, Edinburgh, Midlothian, United Kingdom; University of Maryland School of Medicine

## Abstract

Pneumonic pasteurellosis, caused by Pasteurella multocida, is a common respiratory infection of ruminants that has major economic and welfare implications throughout the world. Here, we report the annotated genome sequences of seven pathogenic strains of P. multocida that were isolated from cattle in the United Kingdom.

## Abstract (precis)

Pneumonic pasteurellosis, caused by Pasteurella multocida, is a common respiratory infection of ruminants that has major economic and welfare implications throughout the world. Here, we report the annotated genome sequences of seven pathogenic strains of P. multocida that were isolated from cattle in the UK.

## ANNOUNCEMENT

Pasteurella multocida is a pathogenic, facultatively anaerobic, Gram-negative bacterium, classified into 3 subspecies, 5 capsular serogroups, and 16 serotypes, causing a wide range of clinical presentations in various host species. While P. multocida serogroup A is a common bovine nasopharyngeal commensal, it is also a major respiratory pathogen causing severe pneumonia in young calves and shipping fever in weaned, stressed beef cattle, resulting in severe morbidity and mortality. Most P. multocida strains isolated from bovine respiratory disease belong to serogroup A, with A:3 being the most common (1).

Pasteurella multocida strains 618/90 and 619/90 were isolated in 1990 from the lungs of two calves challenged with five field strains isolated from cases of bovine pneumonic pasteurellosis on blood agar plates (2). All sampling was carried out in accordance with the UK Animals (Scientific Procedures) Act 1986 in compliance with Home Office Inspectorate regulations and was approved by the Moredun Animal Welfare and Ethical Review Body. The isolates were confirmed as P. multocida by somatic typing and pulsed-field gel electrophoresis (2). Isolate 619/90 was passaged through another calf to confirm its virulence and generated a new pathogenic isolate, 671/90 (3). Here, we report the genome sequences of strains 618/90 and 619/90, four of the five field isolates (one was not available), and resequencing of 671/90 (4), using Illumina short-read and Oxford Nanopore Technologies long-read sequencing to produce single-contig *de novo* genome assemblies.

Frozen stocks (−80°C in 15% glycerol) of P. multocida strains were streaked onto blood agar base no. 2 with sheep blood (Fisher Scientific), and single colonies were grown aerobically at 37° for 3 h in Oxoid nutrient broth (Fisher Scientific). Genomic DNA was extracted from bacterial pellets using a Nanobind CBB (cells, bacteria, blood) big DNA kit (Circulomics). Genomic DNA libraries were prepared using the Nextera XT library preparation kit (Illumina) and sequenced using the Illumina HiSeq platform using a 250-bp paired-end protocol. Long-read genomic DNA libraries were prepared using a rapid barcoding kit (SQK-RBK004) and sequenced using a FLO-MIN106 flow cell in GridION software release 19.12.6, with live base calling provided by Guppy v3.2.10 (Oxford Nanopore Technologies). Illumina reads were adapter trimmed using Trimmomatic v0.30 with a sliding window quality cutoff of Q15 (5). All trimmed raw data analysis was performed on the Galaxy platform (http://usegalaxy.org.au/) (6). Read quality control was performed using FastQC (Galaxy v0.72+galaxy1) (7) and NanoPlot (Galaxy v1.28.2+galaxy1) (8). Hybrid *de novo* assembly was carried out using the Unicycler pipeline (Galaxy v0.4.8.0) (9). Raw Illumina paired-end reads were mapped to the resultant contigs using BWA-MEM (Galaxy v0.7.17.1) (10) in Simple Illumina mode to further improve the assembly quality. The assembly metrics were calculated using QUAST (Galaxy v5.0.2+galaxy1) (11). Annotations were carried out using the NCBI Prokaryotic Genome Annotation Pipeline v4.12 (12). All genomes were assembled as single circular chromosomes, oriented to start at the gene *dnaA*. Default settings were used for all utilized software packages, unless otherwise stated. The raw read statistics, genome assembly metrics, and accession numbers are shown in Table 1.

**TABLE 1:** Features and accession numbers of genomes of seven P. multocida isolates

	Data for Illumina reads:				Data for MinION reads:						
Strain	No. of reads	SRA accession no.	Coverage (×)		No. of reads	*N*_50_ (bp)	SRA accession no.	Coverage (×)	Genome size (bp)	GC content (%)	Genome accession no.
671/90	350,153	SRR12622402	53.7		4,000	10,376	SRR12622391	33.0	2,277,198	40.34	CP058995
619/90	1,323,187	SRR12622401	96.4		39,649	12,824	SRR12622390	38.9	2,277,197	40.34	CP058994
618/90	391,919	SRR12622396	55.1		34,979	13,345	SRR12622389	40.8	2,277,197	40.34	CP058993
X1053	778,140	SRR12622395	115.6		24,667	11,060	SRR12622400	40.9	2,277,198	40.34	CP059065
X0120	275,738	SRR12622394	42.6		31,554	25,088	SRR12622399	124.1	2,277,198	40.34	CP058992
A0757	585,652	SRR12622393	89.5		22,872	7,893	SRR12622398	36.3	2,277,198	40.34	CP059064
A0419	512,760	SRR12622392	69.6		43,418	20,340	SRR12622397	141.0	2,277,198	40.34	CP058991

### Data availability.

The complete genome sequences and raw reads are available in GenBank under BioProject accession number PRJNA645292.
